# Acupuncture and Related Therapies for Treatment of Postoperative Ileus in Colorectal Cancer: A Systematic Review and Meta-Analysis of Randomized Controlled Trials

**DOI:** 10.1155/2018/3178472

**Published:** 2018-07-29

**Authors:** Yihong Liu, Brian H. May, Anthony Lin Zhang, Xinfeng Guo, Chuanjian Lu, Charlie Changli Xue, Haibo Zhang

**Affiliations:** ^1^Guangdong Provincial Academy of Chinese Medical Sciences, Guangdong Provincial Hospital of Chinese Medicine and The Second Clinical College, Guangzhou University of Chinese Medicine, Guangzhou, China; ^2^China-Australia International Research Centre for Chinese Medicine, RMIT University, Bundoora, VIC 3083, Australia

## Abstract

Delays in recovery of intestinal function following abdominal surgery are associated with longer hospital stays, increased postoperative complications, and higher costs to the health care system. Studies of acupuncture for postoperative ileus and other postoperative issues have reported improvements. This systematic review and meta-analysis aimed to assess whether acupuncture assisted recovery following surgery for colorectal cancer (CRC). Randomized controlled trials (RCTs) were identified from major English and Chinese language biomedical databases. Participants (aged 18 years plus) had received surgical resection for CRC. 22 studies (1,628 participants) were included. Five were sham-controlled. Outcomes included gastrointestinal function recovery (21 studies), recovery of urinary function (1 study), postoperative abdominal distension (3 studies), and quality of life (1 study). Meta-analyses found significant reductions in time to first bowel sounds, first flatus, and first defecation in both the sham-controlled and nonblinded studies. These results suggested that the addition of acupuncture following CRC surgery improved recovery of gastrointestinal function based on four blinded good quality RCTs (281 participants) and 17 nonblinded lower quality RCTs (1,265 participants). The best available evidence was for interventions that included electroacupuncture at the point ST36* Zusanli* and there is supporting evidence for other types of acupuncture therapies that involve stimulation of this point. This review is registered with the following: systematic review registration in PROSPERO: CRD42017079590.

## 1. Introduction

Delay in resumption of intestinal function following the surgery occurs in most patients after abdominal surgery including surgery for CRC [1]. Known as postoperative ileus (POI), this condition typically resolves by day five following open abdominal surgery and by day three following laparoscopic surgery but it may be prolonged or recur and may be accompanied by abdominal distension, pain, and/or nausea and vomiting [2]. POI is associated with longer hospital stays and increased postoperative complications and results in higher costs to the health care system [1, 3].

Integrative medicine is utilised by a substantial proportion of people with cancers [4, 5] with acupuncture being used in both out-patient [6] and in-patient settings [7, 8]. A review of acupuncture for symptom management in cancer found evidence of benefits [9] and an update found support for improvement in POI [10]. A meta-analysis of acupuncture for POI found that acupuncture might be effective in improving POI [11] and a systematic review of acupuncture for recovery after CRC surgery concluded that there was low-to-moderate quality evidence for the efficacy and safety of acupuncture for postoperative outcomes including POI [12]. Since these reviews were published, additional studies have become available.

The objective of this systematic review was to assess whether acupuncture and related therapies were effective in assisting in recovery following surgery for colorectal cancer. If so, we aimed to determine the best available evidence in order to inform clinical practice.

## 2. Method

Searches were conducted of (1) major English language biomedical databases: PubMed, Embase, CINAHL, AHMED, and Cochrane Library; (2) major Chinese language biomedical databases: Chinese BioMedical Literature Database (CBM), VIP Database for Chinese Technical Periodicals (CQVIP), China National Knowledge Infrastructure (CNKI), and Wanfang Data from their respective inceptions to October 2017; and (3) reference lists in studies and reviews (see Supp.  1 for PubMed search strategy). Only prospective randomized controlled trials (RCTs) were included.

Included participants had been diagnosed with colorectal, colon, or rectal cancer by pathology and had received surgical resection for this cancer and were aged 18 years or older. Studies that included participants with other cancers or other diseases were excluded.

The test interventions were acupuncture and related therapies including electroacupuncture, manual acupuncture, acupressure, moxibustion, point application and laser acupuncture, or any combination of these therapies. Studies of acupoint-injection, suture embedding (also called “catgut” embedding), transcutaneous electrical nerve stimulation (TENS), acupuncture combined with oral herbal medicine, or other nonacupuncture-related therapy were excluded. Studies in which the details of the acupuncture or related therapy were unclear were excluded.

The control interventions were sham/placebo acupuncture or related therapy, or no additional intervention. The cointerventions were any conventional surgery for CRC, including open or laparoscopic surgery, plus conventional postoperative or perioperative care. The conventional care was required to be the same in all groups. The study setting could be a hospital or similar clinic suitable for postsurgical recovery.

Studies that reported numerical data for an outcome directly related to recovery from CRC surgery were included. Outcomes included recovery of physical functions, quality of life, and postoperative adverse events. Studies of acupuncture for anaesthesia or pain relief were excluded.

Search results were screened by two reviewers and full-text papers were obtained for any paper considered a potential inclusion. These were assessed against the inclusion and exclusion criteria. Data were extracted to a predesigned spread-sheet for: citation details, year, country; study design, duration, and setting; methodological aspects; participant characteristics (number, age, gender, and cancer type); details of the acupuncture or related intervention, type of surgery, type of conventional care; details of outcome measures; data for included outcome measures; safety, dropouts, and adverse events for each group. If there were any disagreements between reviewers, a third reviewer was consulted. In the case of discrepancies in the published data it was planned to contact authors but this was not required. Risk of bias was assessed using the Cochrane tool [13] by two reviewers independently with a third reviewer available or consultation to resolve any issues.

Assessments of effect sizes were based on published data and conducted in Stata 12. Meta-analyses were conducted when studies were comparable and used the same outcome measures. Random-effects models with 95% confidence interval (CI) were applied. Heterogeneity was quantified as I-square. Publication bias was assessed using a funnel plot and Egger's test for asymmetry when ten or more studies were available. Subgroup analyses were planned based on participant characteristics such as the cancer type (colorectal, rectal, and colon); the type of acupuncture or related intervention; the type of surgery (open, laparoscopic); the type of conventional care; and methodological quality. Sensitivity analyses were planned to explore sources of heterogeneity.

## 3. Results

**Figure 1:**
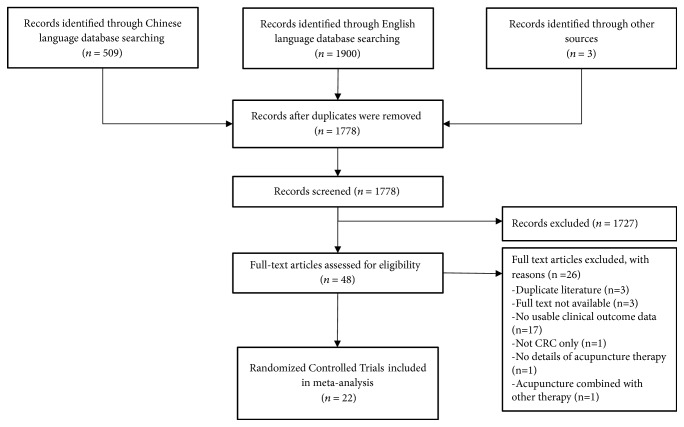
Flow diagram of the search, screening, and inclusion process.

Twenty-two RCTs of acupuncture and related therapies for recovery following surgery for colorectal cancer were identified (Figure 1). One study was conducted in the USA [14], 19 in various locations in mainland China [15–33], one in Hong Kong [34, 35], and one in Taiwan [36].

The studies enrolled 1,628 participants ranging in age from 22 to 87 years (Table 1, study IDs 1-22). The mean ages ranged from 45 to 73 years. In the twenty-one studies that reported data on gender, there were 843 males and 625 females. All participants were diagnosed with CRC in 15 studies, with colon cancer in three studies, and rectal cancer in four studies. Eleven reported that acupuncture sensation (*deqi*) was produced but none mentioned Chinese medicine syndrome differentiation [37].

In two studies laparoscopic surgery was used [27, 34]. One used Dixon surgery for rectal cancer [15] and one used Miles surgery for rectal cancer [27]. The other studies used conventional open surgery, excluded Miles surgery, or did not specify the type of surgery. Two studies used the Fast Track Program (FTP) of perioperative care [22, 24] and the other 20 studies used conventional postoperative care. Two studies involved three intervention groups [25, 34]. One of these had two test groups [25], so whenever both groups were included in the same pool the number in the control group was halved to avoid double counting.

Test interventions included manual acupuncture (3 studies), electroacupuncture (7 studies), manual acupuncture plus electroacupuncture (1 study), acupressure (2 studies), manual acupuncture plus moxibustion (1 study), warm needling (1 study), ear acupressure (3 studies), acupuncture plus ear acupressure (1 study), moxibustion plus ear acupressure (1 study), acupuncture plus electroacupuncture plus ear acupuncture (1 study), and point application (1 study).

Outcome data were available for gastrointestinal function recovery (21 studies), recovery of urinary function (1 study) [27], postoperative abdominal distension (3 studies) [22, 24, 25], and quality of life (1 study) [18].

### 3.1. Risk of Bias

All studies were described as “randomized” but only 13 described an appropriate method for sequence generation and were judged “low risk” while three studies were judged “high risk” since patient order was used (Supp.  Table S1). Four studies described the method of allocation concealment and were judged “low risk”. The others were judged “unclear risk” since there was no description.

Five studies used a sham intervention. Four of these were judged “low risk” for blinding of participants and one was judged “unclear risk” since the method was not well described. All studies were judged “high risk” for blinding of study personnel since the acupuncturists could not be blinded. Five studies were judged “low risk” for blinding of outcome assessors and the other studies were “high risk”. In each study there were no dropouts or few dropouts so all were assessed as “low risk” of bias for incomplete outcome data. Study protocols were located for two studies [14, 34]. Since all outcomes were reported, both these studies were judged “low risk” for selective outcome reporting. The other studies were judged “unclear risk”.

Funnel plots for time to first bowel sounds, time to first flatus, and time to first defecation were generated for acupuncture and related therapies versus conventional care alone. These showed no apparent asymmetry and the Eggers tests were not significant (Supp. Figures S2, S4, and S6). This indicated that the risk of publication bias was not high.

### 3.2. Recovery of Gastrointestinal Function

Of the 21 studies that reported results for recovery of gastrointestinal function, five included comparisons with a sham acupuncture therapy and 17 studies compared the acupuncture therapy with a no acupuncture group. All groups used a form of usual postoperative or perioperative care.

#### 3.2.1. Acupuncture Therapy versus Sham Acupuncture Therapy

All five studies were of CRC. One study used laparoscopic surgery [34] and four used open surgery. All used usual postoperative care in both groups. In two studies the test intervention was electroacupuncture [32, 34]. One study used manual acupuncture plus electroacupuncture plus ear acupuncture [14], one study used acupressure on traditional points [36], and one used point application with warming cataplasm [29]. The most commonly used traditional acupuncture points were ST36* Zusanli* 足三里 (n=5), SP6* Sanyinjiao* 三阴交 (n=2), and LI4* Hegu* 合谷 (n=2). The single ear point was TF4* Shenmen* 神门.

The sham interventions included

- disabled electrostimulator [14, 32, 34];
- needles taped on the same points with no insertion [14];
- needles inserted subcutaneously at a sham point superior and lateral to the verum point [32];
- shallow insertion using short needles, 15mm away from the verum acupoints, with avoidance of acupuncture sensation “*deqi*” [34];
- acupressure using the same method on a nonpoint [36];
- point application with nonwarming cataplasm [29].

**Figure 2:**
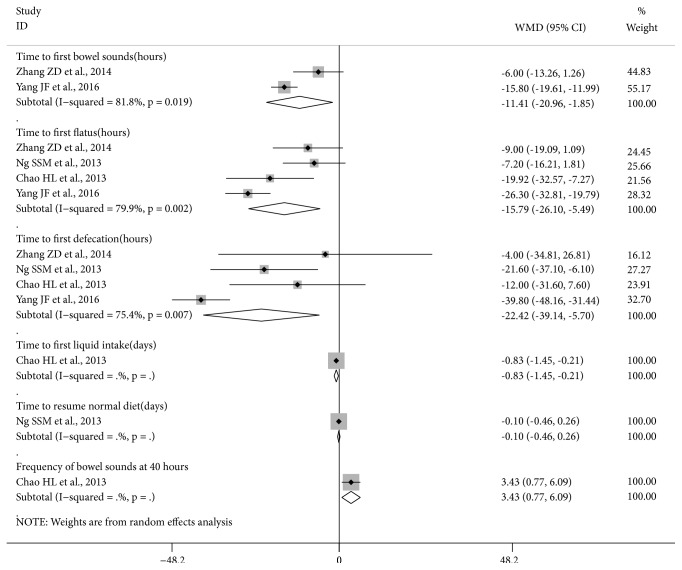
**Forest plot of acupuncture therapy versus sham acupuncture therapy for recovery of gastrointestinal function**. Note: frequency of bowel sounds at 40 hours: frequency per minute assessed during a three-minute interval.

All studies reported data for recovery of gastrointestinal function but one study used composite measures which are reported separately [14]. Data could be pooled for time to first bowel sounds (2 studies), first flatus (4 studies), and first defecation (4 studies) (Figure 2). The other outcomes were reported by single studies only.

There was a significant reduction in time to first bowel sounds in the pooled result for two studies (MD -11.41 [-20.96, -1.85] hours, I^2^=81.8%) but the heterogeneity was considerable (Table 2). For time to first flatus, there were significant reductions in the pool of two studies of electroacupuncture (MD -8.00 [-14.72, -1.28] hours, I^2^=0%) without heterogeneity and in the total pool of four studies (MD -15.79 [-26.10, -5.49] hours, I^2^=79.9%) with considerable heterogeneity. For time to first defecation, the pooled results showed significant reductions for electroacupuncture (MD -18.04 [-31.90, -4.19] hours, I^2^=0.1%) and all acupuncture therapies (MD -22.42 [-39.14, -5.70] hours, I^2^=75.4%). The source of heterogeneity in the previous two total pools was the study of point application therapy [29]. When removed, the meta-analysis results were significant for time to first flatus (MD -10.96 [-17.98, -3.94] hours, I^2^=26.7%) and time to first defecation (MD -16.03 [-27.34, -4.73] hours, I^2^=0%) without important heterogeneity.

In the study of acupuncture plus electroacupuncture plus ear acupuncture [14], the two composite measures were GI-3 (the later of the following two events: time that the patient first tolerated solid food, AND time that the patient first passed flatus OR a bowel movement) and GI-2 (the later of the following two events: time patient first tolerated solid food AND time patient first passed a bowel movement). There were no significant differences between groups for GI3 (MD 3.00 [-26.12, 32.12] hours, n=81) or GI2 (MD -3.00 [-31.74, 25.74] hours, n=81) but the CI were very wide.

#### 3.2.2. Acupuncture Therapy versus Postoperative Care Alone

Seventeen RCTs compared acupuncture plus postoperative care to postoperative care without acupuncture. In two of these studies [22, 24] the Fast Track Program (FTP) of perioperative care was used in both groups.

The most commonly used traditional acupuncture points were ST36* Zusanli* 足三里 (n=14), ST37* Shangjuxu* 上*巨虚* (n=10), LI4* Hegu* 合谷 (n=5), SP6* Sanyinjiao* 三阴交 (n=4), PC6* Neiguan* 内关 (n= 3), and TE6* Zhigou支沟* (n=3). Notably, ST36* Zusanli* 足三里 was included in all studies except the three that used ear acupressure alone. The most frequently used ear points were CO4* Wei* 胃 (n=5), CO7* Dachang* 大肠 (n=4), and CO6* Xiaochang* 小肠 (n=4). In one study, sensitive points on the ear were chosen on an individual basis [21].

**Figure 3:**
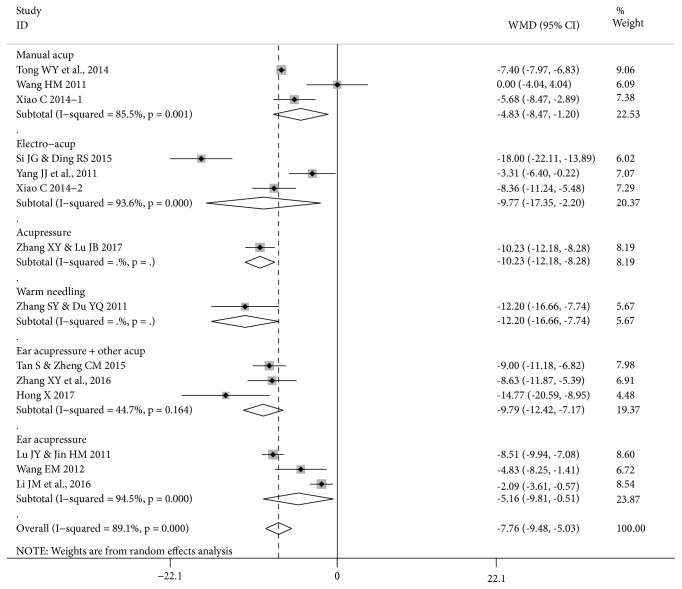
**Forest plot of acupuncture therapy versus postoperative care for time to first bowel sounds (13 RCTs, 14 groups)**. Note: this forest plot focuses on the effect sizes for each study. Since Xiao C 2014 is a three-group study with two test groups of n=30 and one control group n=30, in the meta-analysis in Table 3 the number of participants was halved n=15 in each comparison. In this figure, the control group remains n=30, so this has a small effect on the result for the total pool. The pooled result in Table 3 is the more accurate estimate of the pooled effect size, while the effects for Xiao C 2014-1 and Xiao C 2014-2 are accurate in this figure.

**Figure 4:**
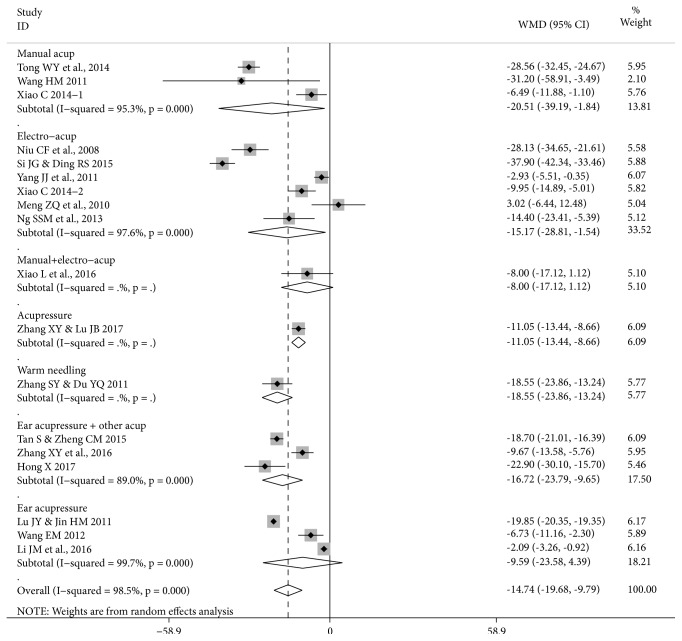
**Forest plot of acupuncture therapy versus postoperative care for time to first flatus (17 RCTs, 18 groups)**. Note: this forest plot focuses on the effect sizes for each study. Since Xiao C 2014 is a three-group study with two test groups of n=30 and one control group n=30, in the main meta-analysis (Table 4) the number of participants was halved n=15 in each comparison. In this figure, the control group remains n=30, so this has a small effect on the result for the total pool. The pooled result in Table 4 is the more accurate estimate of the pooled effect size, while the effects for Xiao C 2014-1 and Xiao C 2014-2 are accurate in this figure.

**Figure 5:**
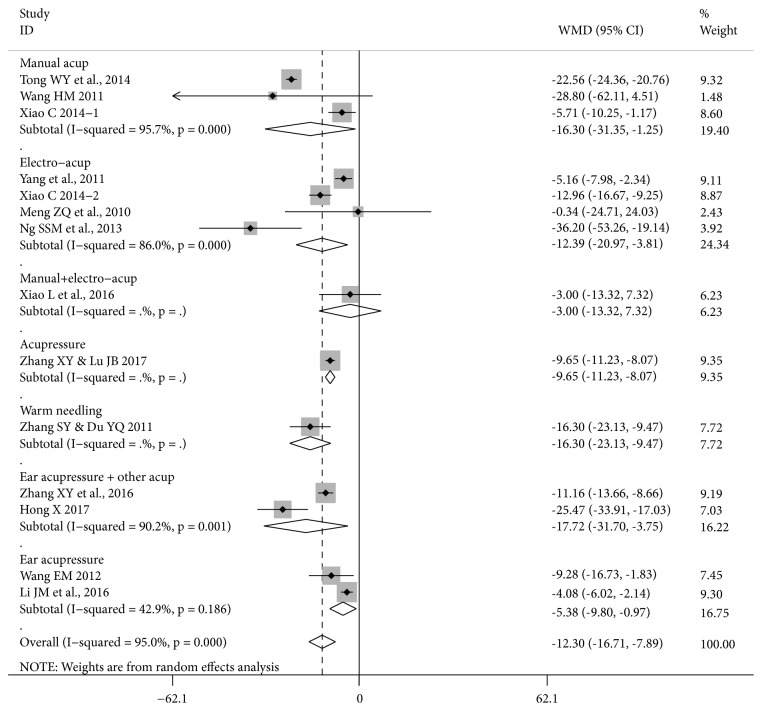
**Forest plot of acupuncture therapy versus postoperative care for time to first defecation (13 RCTs, 14 groups)**. Note: this forest plot focuses on the effect sizes for each study. Since Xiao C 2014 is a three-group study with two test groups of n=30 and one control group n=30, in the meta-analysis in Table 5 the number of participants was halved n=15 in each comparison. In this figure, the control group remains n=30, so this has a small effect on the result for the total pool. The pooled result in Table 5 is the more accurate estimate of the pooled effect size, while the effects for Xiao C 2014-1 and Xiao C 2014-2 are accurate in this figure.

Meta-analysis results are presented separately for time to first bowel sounds, first flatus, and first defecation (Figures 3, 4, and 5 and Tables 3, 4, and 5). Studies of a commonly used acupuncture therapy on traditional points were grouped together, with separate results presented for manual acupuncture, electroacupuncture, manual plus electroacupuncture, acupressure, and warm needling. Studies that combined traditional points with ear points (3 studies) were included as a subgroup within this traditional points group, since this approach is typical of modern acupuncture practice [38]. Ear acupressure alone was treated as a separate group since no traditional points were used.

*Time to First Bowel Sounds. *There were significant reductions in time to first bowel sounds (hours) in the pooled results for studies of manual acupuncture, electroacupuncture, and acupuncture/moxibustion plus ear acupressure and in the single studies of acupressure and warm needling (Figure 3).

The pool of 10 studies of acupuncture or acupressure on traditional points showed a mean reduction of 8.61 hours in the test groups (MD -8.61 [-10.60, -6.61] I^2^=84.8%) but the heterogeneity was considerable (Table 3). Therefore the following sensitivity analyses were conducted. Since FTP has been found to improve recovery [39, 40], it was excluded from the pool. The result for the remaining eight RCTs was significant with reduced heterogeneity (MD -9.73 [-12.21, -7.25] I^2^=80.8%, n=606). Six RCTs in this group were judged low RoB for SG. These also showed a similar result to the total pool (MD -6.95 [-9.90, -4.00] I^2^=82.2%, n=410). The pool of 5 RCTs with low RoB for SG, excluding the remaining study that used FPT, also showed a similar result (MD -8.02 [-10.56, -5.47] I^2^=73.2%, n=380) with reduced heterogeneity. The group for ear acupressure (3 RCTs) showed significant reductions in time to first bowel sounds with considerable heterogeneity. Due to differences between studies there were no reasonable approaches to sensitivity analyses for these groups.

The total pool of 13 RCTs showed a significant reduction with considerable heterogeneity (89.0%). In total, 7 RCTs were judged low RoB for SG. These showed a similar result to the total pool (MD -6.69 [-9.34, -4.04] I^2^=80.5%, n=470). When the remaining study that used FPT also was excluded, the pool of 6 RCTs with low RoB for SG showed a similar result to the total pool with reduced heterogeneity (MD -7.57 [-9.92, -5.21] I^2^=73%, n=440).

*Time to First Flatus. *Seventeen RCTs reported data on time to first flatus (hours) (Figure 4). There were significant reductions in the studies of manual acupuncture and electroacupuncture and in the pooled result for 14 studies that used an acupuncture therapy on traditional points (MD -15.93 [-21.44, -10.41] I^2^=95.5%, n=967) but the heterogeneity was considerable (Table 4).

In the sensitivity analysis of 12 RCTs, after excluding the two studies that used FTP, the result was similar (MD -14.47 [-20.06, -8.88] I^2^=95.3%, n=853). In the pool of nine RCTs judged low RoB SG the result remained significant (MD -9.28 [-13.12, -5.44]) I^2^=80.8%, n=655) with reduced heterogeneity. When the remaining study that used FTP was also excluded, the result for the remaining eight RCTs was similar (MD -8.90 [-12.72, -5.09] I^2^=81.9%, n=625). In the three RCTs of ear acupressure without any traditional points, the pooled result showed a significant reduction. In the total pool of all 17 RCTs, the result were similar to that for the traditional points group with considerable heterogeneity (I^2^=98.4%).

In the sensitivity analyses for the total pool, removing the two studies that used FTP produced a similar result (MD -13.49 [-18.70, -8.29] I^2^=98.6%, n=1151). In the 10 RCTs judged low RoB SG, there was reduced heterogeneity (MD -8.97 [-12.40, -5.55] I^2^=78.9%, n=715) and when the study that also used FTP was excluded, the pooled result for the remaining 9 RCTs was similar (MD -8.67 [-12.06, -5.27] I^2^=79.9%, n=685).

*Time to First Defecation. *There were significant reductions in time to first defecation in the studies of manual acupuncture and electroacupuncture (Figure 5) and in the pooled result of 11 RCTs of acupuncture therapies that used traditional points (MD -13.53 [-18.38, -8.67] I^2^=94.1%, n=850) but the heterogeneity was substantial (Table 5).

In the sensitivity analyses, removal of the two studies that used FTP reduced the heterogeneity (MD -11.31 [-14.58, -8.04] I^2^=79.2%, n=736) and heterogeneity was further reduced in the pool of nine studies judged low RoB SG (MD -10.07 [-12.99, -7.15] I^2^=71%, n=656).

In the total pool of all 13 studies of an acupuncture therapy, the result was significant with substantial heterogeneity (MD -12.34 [-16.84, -7.84] I^2^=94.9%). This heterogeneity was reduced when the two FTP studies were removed (MD -10.29 [-13.31, -7.27] I^2^=83.8%, n=926) and was further reduced in the pool of 10 RCTs with low RoB SG (MD -9.97 [-12.69, -7.25] I^2^=67.7%, n=716).

*Other Measures of Gastrointestinal Recovery. *In addition to the above outcomes, four studies reported on other measures of gastrointestinal recovery (Table 6). In the pool of two studies of CRC that reported time to first liquid intake [17, 21], there was significant improvement in the acupuncture therapy group (MD -19.72 [-20.22, -19.22] I^2^=0%). For time to first semifluid food intake, a single study of colon cancer in elderly patients found no difference between groups [26]. For time to resume normal diet, a single study of laparoscopic surgery for CRC found a significant improvement in the electroacupuncture group [34].

### 3.3. Other Postoperative Recovery Outcomes

One study of manual acupuncture plus moxibustion [27] that reported the incidence of postoperative urinary retention found one case in the acupuncture group and two cases in the usual care group. There was no significant difference between groups (RR 0.50 [0.05, 4.94] n=30).

Three studies reported on abdominal distension at five days after surgery. Since each study used a different approach to reporting data, data were not pooled. One study included two test groups [25] (Table 7). One of the studies that used FTP showed no difference between groups while the others found significant reductions in the groups that received acupuncture.

One study of electroacupuncture (n=76) reported on quality of life using a modified Edmonton Symptom Assessment System (ESAS) which consists of five items (pain, nausea, insomnia, abdominal distension, and general sense of well-being), which are each rated using a 0-10 numeric rating scale. The authors reported no differences between groups for any outcome [18].

### 3.4. Safety

One RCT [18] mentioned that there were no AEs greater than CTCAE grade I [41] for electroacupuncture. Another RCT stated there was no AE for manual acupuncture [27]. The other studies did not mention AEs. Therefore it was not possible to make an assessment of the safety of the acupuncture therapies.

### 3.5. Post Hoc Analyses

It is plausible that acupuncture may show a dose-response effect which is influenced by the type of acupuncture, the number of points needled, the patient response in terms experiencing typical acupuncture sensations (*deqi*), needle retention time, stimulation method, number and frequency of treatments, and other factors [42, 43]. In order to explore this issue we conducted post hoc analyses of the acupuncture therapy versus usual postoperative care group based on available data for the number of acupuncture points used and whether the study mentioned the experience of* deqi*.

There was variation in the number of studies that reported on the three main outcomes with time to first flatus providing the most complete data. For analysis of number of points we selected the subgroup of studies that used acupuncture (manual or electro-), acupressure, and/or moxa on traditional points excluding studies that also used ear points. In this group of 11 studies, which all reported time to first flatus, the number of traditional acupuncture points used ranged from four to 12 (one point used bilaterally was counted as two points), so studies were divided into two groups: 1. 4-6 points; and 2. 8-12 points, which was the most equal division. For each of the three main POI outcomes, both groups showed significant reductions in time to outcome, and the effect sizes were larger for the 8-12 points groups (Supp.  Table S2). However the confidence intervals were overlapping and heterogeneity was substantial to considerable. So while the results suggest a dose-response trend, this could not be confirmed.

For experience of* deqi,* all 11 studies that mentioned that the patient experienced acupuncture sensation(s) were treated as a subgroup irrespective of the points or acupuncture therapy used (Supp.  Table S2). For each of the three main POI outcomes there were significant improvements in the acupuncture therapy groups. The effect sizes were comparable with the results for the total pools and the confidence intervals overlapped. Heterogeneity was reduced but remained substantial to considerable.

## 4. Discussion

Of the 21 studies that reported results for recovery of gastrointestinal function, all but the three studies of ear acupressure alone used the point ST36* Zusanli* 足三里, usually in combination with other points. In the case of the ear acupressure alone studies, these all used CO4* Wei* 胃 and CO7* Dachang* 大肠 plus other points. Each of these points is commonly used for gastrointestinal disorders [38, 44]. Of the stimulation methods, manual acupuncture with or without electrostimulation was the most frequently used. Other commonly used methods included acupressure, moxibustion, and ear acupuncture or acupressure [38, 44]. Overall, this group of studies was reflective of the scope of acupuncture practice internationally.

For the five blinded sham-controlled studies, data pooling was feasible for four studies and for the three main POI outcomes. All these outcomes showed significant reductions in the time to these events. The heterogeneity in the total pools was attenuated by removal of the single study of point application therapy without affecting the overall result. There was no apparent effect on the result for the type of sham used. This was consistent with earlier reviews [45, 46].

In the remaining 17 studies, there were significant differences in the pooled data in favour of the acupuncture and related therapy groups compared to the conventional care alone groups for all three main measures of gastrointestinal function recovery but the heterogeneity was considerable in the pooled results. Heterogeneity was still evident when data were grouped according to the type of acupuncture intervention, while the significant differences were maintained. Removal of the studies that used FTP, which is likely to have provided an independent contribution to recovery, reduced heterogeneity somewhat but there were only two such studies so the reductions were small. The sensitivity analyses of studies that were judged low RoB SG showed lower heterogeneity and some reductions in effect sizes but the heterogeneity remained moderate to substantial. Further attempts to group studies by combinations of surgery type, cancer type, and acupuncture type were not productive since any resultant pools comprised too few studies. It is notable that the direction of the effect in the majority of studies was in favour of the acupuncture interventions. So it appears likely that the statistical heterogeneity reflected the clinical diversity amongst the studies and variation in effect sizes. It was not an indicator of an unclear direction in the results. In addition to measures of POI, acupuncture appeared to reduce postoperative abdominal distension but there were too few studies for any strong conclusions.

Overall, the results of the 21 RCTs indicated that acupuncture therapies reduced time to recovery of gastrointestinal function following CRC surgery. In studies of other abdominal surgeries, acupuncture has been reported to reduce POI following gastrectomy [47, 48] and caesarean section [49], so the effects found in this meta-analysis are not limited to surgery for CRC. With regard to type of surgery, most data were for open surgery but significant reductions in time to recovery were also evident in studies of laparoscopic surgery for CRC and surgery for rectal cancer.

With regard to type of acupuncture therapy, the sham-controlled studies which constitute the better quality evidence support electroacupuncture on the point ST36* Zusanli* 足三里, with or without other points, as effective for recovery of POI. In the nonblinded studies, the heterogeneity in meta-analysis pools precluded detailed assessment of which type of acupuncture was more effective. The results appear to support various types of acupuncture therapy on ST36* Zusanli* 足三里 combined with other points. In general, the categories of acupuncture were not obviously different from each other in terms of effect sizes. Notably, electroacupuncture did not appear to be any better than manual acupuncture. Also, the pooled effect size results for the sham-controlled studies were comparable with those for the nonblinded studies. This suggests that lack of blinding did not lead to inflation of effect sizes. It is notable that all the studies were conducted in a hospital setting, so it is likely that there was little opportunity for participants to interact with each other over the short durations of the studies, and the outcome data were usually collected by nursing staff. These features of the setting appear conducive to the collection of more objective data than may be the case in longer studies in out-patient settings.

A previous meta-analysis of acupuncture for headache suggested that electroacupuncture was more effective than manual acupuncture, longer needle retention was better, and twice-a-week treatment was better than once-a-week treatment [50], a review of acupuncture for menstrual pain found effects for needle location, number of needles used, and frequency of treatment [51], but a clinical study found no significant effects of needle retention duration on outcomes in oncology [52]. In attempting to determine whether there was any dose-response effect for the acupuncture on POI outcomes, the only feasible parameters were overall number of points used and patient experience of* deqi*. It was not possible to determine estimates of the total number of treatments or total duration of treatment since treatment typically ceased once the outcome had been achieved. The results suggested that more points may be better but the wide confidence intervals and statistical heterogeneity precluded any strong conclusions. There were too few studies for any effects of number of ear points to be examined. Future studies could consider designs that directly test potential dose-related factors.

Patient experience of* deqi* did not appear to affect results. Since* deqi *is a typical aspect of acupuncture practice, it is likely that mention was omitted in a number of study reports. Therefore, the meaningfulness of this result is unclear. Notably, the sham-controlled studies of acupuncture, which were reported in more detail, did mention* deqi*.

One limitation with these meta-analyses was the methodological quality and associated risk of bias in the included studies. The majority of the studies were not blind to participants and the acupuncturists were not blinded in any of the studies. Nevertheless, the results of the multiple sham-controlled studies tended to agree with those of the nonblinded studies. Many studies conducted in China do not report according to the CONSORT or STRICTA guidelines, resulting in omission of important aspects of trial methodology [37, 53]. From the point of view of meta-analysis, inadequacies in study reporting substantially limit the opportunities for exploration of clinically relevant variables [54], so it is vital that journals endorse these guidelines.

Although most of the studies were conducted in China, integrative cancer therapy employing acupuncture is used in hospitals outside China and has proven acceptable to patients in Europe, America, and Australia [52, 55]. Based on the results of this meta-analysis, the extension of acupuncture use in postoperative care should be considered.

## 5. Conclusions

The addition of an acupuncture intervention following surgery for CRC improved outcomes for recovery of gastrointestinal function based on pooled data from four blinded good quality RCTs (281 participants) and 17 nonblinded lower quality RCTs (1,265 participants). The best available evidence was for interventions that included electroacupuncture at the point ST36* Zusanli.* There is supporting evidence for other types of acupuncture therapies that involve stimulation of this point plus other points. Further well-designed blinded studies are needed to confirm these findings and determine optimal acupuncture interventions for POI.

## Figures and Tables

**Table 1:** Characteristics of included studies of acupuncture and related therapies for postoperative recovery for colorectal cancer.

**Study ID No. [ref]**	**Author, year [location]**	**N participants (baseline); mean (SD) age at baseline in years; (M/F); **	**N groups; Duration and frequency of treatment **	**Conventional treatment, cancer type**	**Acupuncture therapy**	**Control intervention**	**Acupuncture points**
1 [36]	Chao HL *et al*., 2013 [3]	66; T: 61.9(13.3), C: 62.8(15.7); (31/35)	2; from postoperative days 1-5, three-mins, three times per day	Open surgery, CRC	Acupressure*∗*	Sham acupressure	ST36 *Zusanli* 足三里, NS

2 [14]	Deng G *et al*., 2013 [2]	90; T: 56, C: 59; (52/38)	2; from postoperative days 1-3, 30 min, twice daily	Open surgery, CRC	Acupuncture + electro-acupuncture + ear acupuncture	Sham acupuncture + sham electro-acupuncture + sham ear acupuncture	ST36 *Zusanli *足三里, PC6 *Neiguan *内关, LI4 *Hegu *合谷, SP6 *Sanyinjiao *三阴交, SP9 *Yinglingquan *阴陵泉, ST25 *Tianshu *天枢 and ear point TF4 *Shenmen *神门, bilateral

3 [15]	Hong X 2017 [1]	80; T: 61.2(7.2), C: 58.8(9.4); (47/33)	2; ear acupressure: press Vaccaria seeds 5 times per day for 7 days, change to other ear every 2 days; moxibustion: once a day for 7 days	Dixon surgery, rectal cancer	Moxibustion + ear acupressure	no acupuncture therapy	Moxibustion points: ST36 *Zusanli* 足三里 and CV12* Zhongwan* 中脘; ear points: CO4 *Wei* 胃, TF4 *Shenmen* 神门, CO6 *Xiaochang* 小肠, CO3 *Benmen贲门*, unilateral

4 [16]	Li JM *et al*., 2016 [1]	160; T: 51.3(3.16), C: 52.17(3.34)	2; press Vaccaria seeds 30-60s per point, three-five times per day, change to other ear every 3-7 days	Open radical surgery, rectal cancer	Ear acupressure	no acupuncture therapy	Ear points: CO4 *Wei* 胃, CO5 *Shierzhichang* 十二*指肠*, CO6 *Xiaochang *小肠,CO7 *Dachang* 大肠, CO13 *Pi 脾*, CO3 *Benmen贲门*, unilateral

5 [17]	Lu JY & Jin HM 2011 [1]	78; T: 62.25(9.38), C: 61.21(10.16); (43/35)	2; Vaccaria seeds attached immediately after surgery, point pressure for 30-60s per point every 2-3 hours (more than 5 min/cycle); until recovery	Radical surgery excluding Miles surgery, CRC	Ear acupressure*∗*	no acupuncture therapy	Ear points: CO13 *Pi 脾*, CO4 *Wei* 胃, CO7 *Dachang* 大肠, CO6 *Xiaochang* 小肠, AH6a *Jiaogan交感*, CO18 *Neifenmi* 内*分泌*, AT4 *Pizhixia 皮质*下, bilateral

6 [18]	Meng ZQ *et al*., 2010 [1]	85; T: 54.3, C: 53.1; (47/38)	2; from postoperative days 1-6, 20 min, once a day, until first bowel movement or day 6, whichever came first	Intraperitoneal surgery, epidural anaesthesia, colon cancer	Electro-acupuncture	no acupuncture therapy	ST36* Zusanli* 足三里, ST37 *Shangjuxu* 上*巨虚*, TE6 *Zhigou支沟*, GB34 *Yanglingquan *阳陵泉, bilateral

7 [34, 35]	Ng SSM *et al*., 2013 [4] †	165; T: 67.4(9.7), C1: 67.4(10.7), C2:68.5(10.6); (99/66)	3; T & C1: from postoperative days 1-4, 20 min, once a day, until defaecation occurred or day 4, whichever was earlier	Laparoscopic surgery, CRC	Electro-acupuncture*∗*	C1: sham electro-acupuncture, C2: no acupuncture therapy	ST36 *Zusanli *足三里, SP6 *Sanyinjiao *三阴交, LI4 *Hegu *合谷, TE6* Zhigou支沟*, NS

8 [19]	Niu CF *et al*., 2008 [1]	32; T & C: 52; (21/11)	2; from postoperative day 1, 15 min. twice daily	Radical surgery excluding Miles surgery, CRC	Electro-acupuncture	no acupuncture therapy	ST36 *Zusanli* 足三里, ST37* Shangjuxu *上*巨虚*, PC6* Neiguan* 内关, NS

9 [20]	Si JG & Ding RS 2015 [1]	40; T: 45, C: 46; (23/17)	2; from 24 hours after surgery, 20 min once a day, till bowel sounds and passing flatus occurred	Radical surgery, CRC	Electro-acupuncture	no acupuncture therapy	ST36* Zusanli *足三里, ST37 *Shangjuxu* 上*巨虚*, SP6 *Sanyinjiao* 三阴交, ST25* Tianshu* 天枢, LI4 *Hegu* 合谷, bilateral

10 [21]	Tan S & Zheng CM 2015 [1]	76; T: 63.8(11.2), C: 63.2(10.5); (43/33)	2; ear acupressure: Vaccaria seeds attached after surgery, pressure for 5 min every 2-3 hours; acupuncture: after surgery, 20 min once a day; until recovery	Radical surgery, CRC	Acupuncture*∗* + ear acupressure*∗*	no acupuncture therapy	ST36 *Zusanli* 足三里 and ear sensitive points *敏感点*, NS

11 [22]	Tong WY *et al*., 2014 [1]	84; T: 58.6(15.1), C: 59.2(14.7); (50/34)	2; from 2 hours after surgery, 30 min, once a day	Open surgery, FTP, rectal cancer	Acupuncture	no acupuncture therapy	ST36 *Zusanli *足三里, PC6 *Neiguan* 内关, ST37 *Shangjuxu* 上*巨虚*, SP4 *Gongsun公孙*, NS

12 [23]	Wang EM 2012 [1]	60; T: 57.4(13.6), C: 56.6(12.3); (34/26)	2; Vaccaria seeds attached 4 hours after surgery, pressure for 1 min on each point, every 6 hours, until recovery	Usual surgery, colon cancer	Ear acupressure*∗*	no acupuncture therapy	Ear points: TF4 *Shenmen* 神门, AH6a* Jiaogan 交感*, AT4 *Pizhixia 皮质*下,CO7* Dachang* 大肠, CO4 *Wei* 胃, *Ashixue 阿*是穴 (sensitive point); unilateral, change side every 4 days

13 [24]	Wang HM 2011 [1]	30; T: 60.4(11.01), C: 58(10.24); (20/10)	2; from 24 hours after surgery, 30 min, once a day, for 5 days	Open surgery, FTP, CRC	Acupuncture	no acupuncture therapy	ST36 *Zusanli *足三里, PC6 *Neiguan* 内关, ST37 *Shangjuxu* 上*巨虚*, SP4 *Gongsun公孙*, NS

14 [25]	Xiao C 2014 [1]	90; T1: 55.87(10.49), T2: 55.33(10.83), C: 54.63(10.25); (51/39)	3; from 24 hours after surgery, 20 min, once a day, for 5 days	Open radical surgery excluding Miles surgery, CRC	T1:acupuncture;*∗* T2: electro-acupuncture	no acupuncture therapy	ST36 *Zusanli *足三里, ST37 *Shangjuxu* 上*巨虚*, bilateral

15 [26]	Xiao L *et al*., 2016 [1]	60; T: 67.43(16.35), C: 68.52(17.16); (33/27)	2; from postoperative days 1-14, 30 min twice daily	Radical surgery, colon cancer in elderly patients	Acupuncture*∗* + electro-acupuncture	no acupuncture therapy	ST36 *Zusanli *足三里, LI4 *Hegu* 合谷, SP6 *Sanyinjiao* 三阴交, LU5 Chize *尺泽* (all electro); TE6* Zhigou支沟*, LU7 *Lieque 列缺* (both manual); all bilateral

16 [27]	Yan YB 2011 [1]	30; T: 50.73(6.076), C: 50.33(5.802); (9/21)	2; from postoperative days 3-7, 30 min, once a day	Laparoscopic Miles surgery, rectal cancer Dukes A-C1, recovery of urinary function	Acupuncture + moxibustion	no acupuncture therapy	ST36 *Zusanli *足三里, LI4* Hegu* 合谷, SP10* Xuehai* 血海, SP9 *Yinglingquan *阴陵泉, SP6* Sanyinjiao* 三阴交, LR3 *Taichong 太冲*, CV4 *Guanyuan关元* (moxa), CV8 *Shenque* 神阙(moxa), NS

17 [28]	Yang JJ *et al*., 2011 [1]	60; T: 60.9(6.63), C: 62(6.98); (39/21)	2; from postoperative day 1, 30 min, once a day, until 3 days after defecation occurred	Radical surgery, CRC	Electro-acupuncture*∗*	no acupuncture therapy	ST36 *Zusanli *足三里, ST37 *Shangjuxu* 上*巨虚*, ST39 *Xiajuxu* 下*巨虚*, bilateral

18 [29]	Yang JF *et al*., 2016 [1]	72; T: 72(5.4), C: 73.4(5.6); (44/28)	2; performed three times at 24 hours, 48 hours and 72 hours after surgery, application for 6 hours each time	Open surgery, CRC in elderly patients	Point application with warming cataplasm	Point application with non-warming cataplasm	ST36 *Zusanli* 足三里, bilateral

19 [30]	Zhang SY & Du YQ 2011 [1]	70; T: 57.1(11.7), C: 59.1(8.5); (52/18)	2; from postoperative days 1-10, 45 min, once a day	Radical surgery, CRC	Warm needling	no acupuncture therapy	ST36 *Zusanli *足三里, ST37 *Shangjuxu* 上*巨虚*, ST39 *Xiajuxu* 下*巨虚*, SP6* Sanyinjiao* 三阴交, SP9 *Yinglingquan* 阴陵泉, NS

20 [31]	Zhang XY & Lu JB 2017 [1]	80; T: 64.8(5.6), C: 67.5(6.0); (42/38)	2; from 6 hours after surgery, 1 min pressure for each point, three times per day, 1 hour after meals, until postoperative day 5	Radical surgery excluding Miles surgery, CRC	Acupressure*∗*	no acupuncture therapy	ST36 *Zusanli* 足三里, LI4 *Hegu* 合谷, ST37 *Shangjuxu* 上*巨虚*, NS

21 [32]	Zhang ZD *et al*., 2014 [1]	40; T: 63(9), C: 60(10); (22/18)	2; at 30 min after surgery, then postoperative days 1–4, 30 min, once a day	Open surgery, CRC	Electro-acupuncture*∗*	Sham electro-acupuncture	ST36 Zusanli 足三里, bilateral

22 [33]	Zhang XY *et al*., 2016 [1]	80; T: 64.8(6.8), C: 67.5(4.2); (41/39)	2; from 6 hours after surgery, 1 min pressure for each point, three times per day, 1 hour after meals, until postoperative day 5, change ear every 2 days	Usual surgery, CRC	Acupressure*∗* + ear acupressure*∗*	no acupuncture therapy	Acupressure points: ST36* Zusanli* 足三里, LI4 *Hegu *合谷, ST37 *Shangjuxu *上*巨虚*, NS; ear points: CO4 *Wei *胃, CO6 *Xiaochang* 小肠, CO7 *Dachang* 大肠, unilateral

Location in which the study was conducted: 1: Mainland China; 2: USA; 3: Taiwan; 4: Hong Kong. NS: side not specified

*∗* means mentioned acupuncture sensation (*deqi*) was obtained. † means reported in two journal articles, reference Nos. [34, 35].

**Table 2:** Acupuncture therapy versus sham acupuncture therapy for recovery of gastrointestinal function.

**Outcome **	**Treatment type, cancer, participants (number)**	**Acupuncture therapy**	**Effect Size MD [95**%** CI] **	**Study ID No.†**
			**I** ^**2**^	
Time to first bowel sounds (hours)	Open surgery, CRC (39)	Electro-acup.	-6.00 [-13.26, 1.26]	21
	Open surgery, elderly, CRC (72)	Point application	-15.8 [-19.61, -11.99]*∗*	18
	**Pooled result (111) 2** ** RCTs**	All acupuncture therapies	-11.41 [-20.96, -1.85]*∗* 81.8%	18, 21

Time to first flatus (hours)	Laparoscopic surgery, CRC (110)	Electro-acup.	-7.20 [-16.21, 1.81]	7
	Open surgery, CRC (39)	Electro-acup.	-9.00 [-19.09, 1.09]	21
	**Pooled result (149)**	Electro-acup.	-8.00 [-14.72, -1.28]*∗* 0%	7, 21
	Open surgery, CRC (60)	Acupressure	-19.92 [-32.57, -7.27]*∗*	1
	Open surgery, elderly, CRC (72)	Point application	-26.30 [-32.81, -19.79]*∗*	18
	**Pooled result (281) 4 ** **RCTs**	All acupuncture therapies	-15.79 [-26.10, -5.49]*∗* 79.9%	1, 7, 18, 21

Time to first defecation (hours)	Laparoscopic surgery, CRC (110)	Electro-acup.	-21.60 [-37.10, -6.11]*∗*	7
	Open surgery, CRC (39)	Electro-acup.	-4.00 [-34.81, 26.81]	21
	**Pooled result (149)**	Electro-acup.	-18.04 [-31.90, -4.19]*∗* 0.1%	7, 21
	Open surgery, CRC (60)	Acupressure	-12.00 [-31.60, 7.60]	1
	Open surgery, elderly, CRC (72)	Point application	-39.80 [-48.16, -31.44]*∗*	18
	**Pooled result (281)4 ** **RCTs**	All acupuncture therapies	-22.42 [-39.14, -5.70]*∗* 75.4%	1, 7, 18, 21

Time to first liquid intake (days)	Open surgery, CRC (60)	Acupressure	-0.83 [-1.45, -0.21]*∗*	1

Time to resume normal diet (days)	Laparoscopic surgery, CRC (110)	Electro-acup.	-0.10 [-0.46, 0.26]	7

Frequency of bowel sounds at 40 hours^1^	Open surgery, CRC (60)	Acupressure	3.43 [0.77, 6.09]*∗*	1

*∗* means statistically significant; † see Table 1; 1: frequency per minute assessed during a three minute interval.

CI: confidence interval; MD: mean difference.

**Table 3:** Acupuncture therapy versus postoperative care for time to first bowel sounds.

**Treatment type, cancer, participants (number)**	**Acupuncture therapy**	**Effect Size MD [95**%** CI] **	**Study ID No.†**
		**hours, I** ^**2**^	
Open surgery, FTP, rectal cancer (84)	Manual acup.	-7.40 [-7.97, -6.83]*∗*	11

Open surgery, FTP,^1^ CRC (30)	Manual acup.	0.00 [-4.04, 4.04]	13

Open radical surgery,^2^ CRC (60)	Manual acup.	-5.68 [-8.47, -2.89]*∗*	14.1 (T1)

**Pooled result (174) 3 RCTs**	Manual acup.	-4.83 [-8.47, -1.20]*∗* 85.5%	11, 13, 14

Radical surgery, CRC (40)	Electro-acup.	-18.00 [-22.11, -13.89]*∗*	9

Radical surgery, CRC (60)	Electro-acup.	-3.31 [-6.40, -0.22]*∗*	17

Open radical surgery,^2^ CRC (60)	Electro-acup.	-8.36 [-11.24, -5.49]*∗*	14.2 (T2)

**Pooled result (160) 3 RCTs**	Electro-acup.	-9.77 [-17.35, -2.20]*∗* 93.6%	9, 14, 17

Radical surgery,^2^ CRC (80)	Acupressure	-10.23 [-12.18, -8.28]*∗*	20

Radical surgery, CRC (70)	Warm needling	-12.20 [-16.66, -7.74]*∗*	19

**Pooled result (454) 7 RCTs**	Manual, electro-, acupressure, warm needling	-8.06 [-10.65, -5.47]*∗* 88%	9, 11, 13, 14, 17, 19, 20

Radical surgery, CRC (76)	Manual acup. plus ear acupressure ^3^	-9.00 [-11.18, -6.82]*∗*	10

Usual surgery, CRC (80)	Acupressure plus ear acupressure	-8.63 [-11.87, -5.39]*∗*	22

Dixon surgery, rectal cancer (80)	Moxa plus ear acupressure	-14.77 [-20.59, -8.95]*∗*	3

**Pooled result (236) 3 RCTs**	Manual acup./ moxa plus ear acupressure	-9.79 [-12.42, -7.17]*∗* 44.7%	3, 10, 22

**Pooled result (690) 10 RCTs**	All acup., acupressure, moxa on trad. points	-8.61 [-10.60, -6.61]*∗* 84.8%	3, 9-11, 13, 14, 17, 19, 20, 22

Radical surgery,^2^ CRC (78)	Ear acupressure	-8.51 [-9.94, -7.08]*∗*	5

Usual surgery, colon cancer (60)	Ear acupressure	-4.83 [-8.25, -1.41]*∗*	12

Open radical surgery, rectal cancer (160)	Ear acupressure	-2.09 [-3.61, -0.57]*∗*	4

**Pooled result (298) 3 RCTs**	Ear acupressure	-5.16 [-9.81, -0.51]*∗* 94.5%	4, 5, 12

**Total pool (988) 13 RCTs**	All acupuncture therapies	-7.78 [-9.55, -6.01]*∗* 89.0%	All above

*∗* means statistically significant; † see Table 1; 1: acupuncture began 24 hours after surgery; 2: excluding Miles surgery; 3: using sensitive ear points.

CI, Confidence Interval; MD, mean difference; moxa, moxibustion; trad., traditional.

**Table 4:** Acupuncture therapy versus postoperative care for time to first flatus.

**Treatment type, cancer, participants (number)**	**Acupuncture therapy**	**Effect Size MD [95**%** CI] **	**Study ID No.†**
		**hours I** ^**2**^	
Open surgery, FTP, rectal cancer (84)	Manual acup.	-28.56 [-32.45, -24.67]*∗*	11

Open surgery, FTP, CRC (30)	Manual acup.	-31.20 [-58.91, -3.49]*∗*	13

Open radical surgery^2^, CRC (60)	Manual acup.	-6.49 [-11.88, -1.10]*∗*	14.1 (T1)

**Pooled result (174) 3 RCTs**	Manual acup.	-20.51 [-39.19, -1.84]*∗* 95.3%	11, 13, 14

Radical surgery^2^, CRC (32)	Electro-acup.	-28.13 [-34.65, -21.61]*∗*	8

Radical surgery, CRC (40)	Electro-acup.	-37.90 [-42.34, -33.46]*∗*	9

Radical surgery, CRC (60)	Electro-acup.	-2.93 [-5.51, -0.35]*∗*	17

Open radical surgery^2^, CRC (60)	Electro-acup.	-9.95 [-14.89, -5.01]*∗*	14.2 (T2)

Intraperitoneal surgery, colon cancer (75)	Electro-acup.	3.02 [-6.44, 12.48]*∗*	6

Laparoscopic surgery, CRC, (110)	Electro-acup.	-14.40 [-23.41, -5.39]*∗*	7

**Pooled result (377) 6 RCTs**	Electro-acup.	-15.17 [-28.81, -1.54]*∗* 97.6%	6-9, 14, 17

Radical surgery, colon cancer, elderly patients (60)	Manual plus electro-acup.	-8.00 [-17.12, 1.12]	15

Radical surgery^2^, CRC (80)	Acupressure	-11.05 [-13.44, -8.66]*∗*	20

Radical surgery, CRC (70)	Warm needling	-18.55 [-23.86, -13.24]*∗*	19

**Pooled result (731) 11 RCTs**	Manual, electro-, acupressure, warm needling	-15.68 [-23.03, -8.33]*∗* 96.1%	6-9, 11, 13-15, 17, 19, 20

Radical surgery, CRC (76)	Manual acup. plus ear acupressure ^3^	-18.70 [-21.01, -16.39]*∗*	10

Usual surgery, CRC (80)	Acupressure plus ear acupressure	-9.67 [-13.58, -5.76]*∗*	22

Dixon surgery, rectal cancer (80)	Moxa plus ear acupressure	-22.90 [-30.10, -15.70]*∗*	3

**Pooled result (236) 3 RCTs**	Manual acup./ moxa plus ear acupressure	-16.72 [-23.79, -9.65]*∗* 89%	3,10, 22

**Pooled result (967) 14 RCTs**	All acup., acupressure, moxa on trad. points	-15.93 [-21.44, -10.41]*∗* 95.5%	3, 6-11, 13-15, 17, 19, 20, 22

Radical surgery^2^, CRC (78)	Ear acupressure	-19.85 [20.35, -19.35]*∗*	5

Usual surgery, colon cancer (60)	Ear acupressure	-6.73 [-11.16, -2.30]*∗*	12

Open radical surgery, rectal cancer (160)	Ear acupressure	-2.09 [-3.26, -0.92]*∗*	4

**Pooled result (298) 3 RCTs**	Ear acupressure	-9.59 [-23.58, 4.39] 99.7%	4, 5, 12

**Total pool (1265) 17 RCTs**	All acupuncture therapies	-14.77 [-19.75, -9.79]*∗* 98.4%	All above

*∗* means statistically significant; † see Table 1; 1: acupuncture began 24 hours after surgery; 2: excluding Miles surgery; 3: using sensitive ear points.

CI, Confidence Interval; MD, mean difference; moxa, moxibustion; trad., traditional.

**Table 5:** Acupuncture therapy versus postoperative care for time to first defecation.

**Treatment type, cancer, participants (number)**	**Acupuncture therapy**	**Effect Size MD [95**%** CI] I**^**2**^	**Study ID No.†**
Open surgery, FTP, rectal cancer (84)	Manual acup.	-22.56 [-24.36, -20.76]*∗*	11

Open surgery, FTP^1^, CRC (30)	Manual acup.	-28.80 [-62.11, 4.51]	13

Open radical surgery^2^, CRC (60)	Manual acup.	-5.71 [-10.25, -1.17]*∗*	14.1 (T1)

**Pooled result (174) 3 RCTs**	Manual acup.	-16.30 [-31.35, -1.25]*∗* 95.7%	11, 13, 14

Radical surgery, CRC (60)	Electro-acup.	-5.16 [-7.98, -2.34]*∗*	17

Open radical surgery^2^, CRC (60)	Electro-acup.	-12.96 [-16.67, -9.25]*∗*	14.2 (T2)

Intraperitoneal surgery, colon cancer (76)	Electro-acup.	-0.34 [-24.71, 24.03]	6

Laparoscopic surgery, CRC (110)	Electro-acup.	-36.20 [-53.26, -19.14]*∗*	7

**Pooled result (306) 4 RCTs**	Electro-acup.	-12.39 [-20.97, -3.81]*∗* 86%	6, 7, 14, 17

Radical surgery, colon cancer, elderly patients (60)	Manual plus electro-acup.	-3.00 [-13.32, 7.32]	15

Radical surgery^2^, CRC (80)	Acupressure	-9.65 [-11.23, -8.07]*∗*	20

Radical surgery, CRC (70)	Warm needling	-16.30 [-23.13, -9.47]*∗*	19

**Pooled result (660) 9 RCTs**	Manual, electro-, acupressure, warm needling	-12.68 [-18.60, -6.77]*∗* 94.8%	6, 7, 11, 13-15, 17, 19, 20

Usual surgery, CRC (80)	Acupressure plus ear acupressure	-11.16 [-13.66, -8.66]*∗*	22

Dixon surgery, rectal cancer (80)	Moxa plus ear acupressure	-25.47 [-33.91, -17.03]*∗*	3

**Pooled result (160) 2 RCTs**	Manual acup./ moxa plus ear acupressure	-17.72 [-31.70, -3.75]*∗* 90.2%	3, 22

**Pooled result (820) 11 RCTs**	All acup., acupressure, moxa on trad. points	-13.53 [-18.38, -8.67]*∗* 94.1%	3, 6, 7, 11, 13-15, 17, 19, 20, 22

Usual surgery, colon cancer (60)	Ear acupressure	-9.28 [-16.73, -1.83]*∗*	12

Open radical surgery, rectal cancer (160)	Ear acupressure	-4.08 [-6.02, -2.14]*∗*	4

**Pooled result (220) 2 RCTs**	Ear acupressure	-5.38 [-9.80, -0.97]*∗* 42.9%	4, 12

**Total pool (1040) 13 RCTs**	All acupuncture therapies	-12.34 [-16.84, -7.84]*∗* 94.9%	All above

*∗* means statistically significant; † see Table 1; 1: acup. began 24 hours after surgery; 2: excluding Miles surgery.

CI, Confidence Interval; MD, mean difference; moxa, moxibustion; trad., traditional.

**Table 6:** Acupuncture therapy versus postoperative care for other measures of gastrointestinal recovery.

**Outcome**	**Treatment type, cancer, participants (number)**	**Acupuncture therapy**	**Effect Size MD** ^**1**^ ** [95**%** CI] **	**Study ID No.†**
			**I** ^**2**^	
Time to first liquid intake (hours)	Radical surgery, CRC (76)	Manual acup. plus ear acupressure^2^	-18.90 [-21.20, -16.60]*∗*	10
	Radical surgery^1^, CRC (78)	Ear acupressure	-19.76 [-20.27, -19.25]*∗*	5

**Pooled result (hours)**	2 RCTs (154)	Manual acup., ear acupressure	-19.72 [-20.22, -19.22]*∗* 0%	5, 10

Time to first semifluid food intake (hours)	Radical surgery, colon cancer, elderly patients (60)	Manual plus electro-acup.	-4.00 [-37.46, 29.46]	15

Time to resume normal diet (days)	Laparoscopic surgery, CRC (110)	Electro-acup.	-0.80 [-1.40, -0.20]*∗*	7

*∗* means statistically significant; † see Table 1; 1: excluding Miles surgery; 2: on sensitive ear points.

CI: confidence interval; MD: mean difference.

**Table 7:** Acupuncture therapy versus postoperative care for postoperative abdominal distension.

**Treatment type, cancer, participants (number)**	**Acupuncture therapy**	**Effect Size MD/RR [95**%** CI] I**^**2**^	**Study ID No.†**
Open surgery, FTP^1^, CRC (30)	Manual acup.	RR 0.67 [0.13, 3.44]^3^	13

Open surgery, FTP, rectal cancer (84)	Manual acup.	MD -0.77 [-0.80, -0.74]*∗*	11

Open radical surgery^2^, CRC (60)	Manual acup.	MD -0.27 [-0.51, -0.03]*∗*	14.1 (T1)

Open radical surgery^2^, CRC (60)	Electro-acup.	MD -0.53 [-0.75, -0.32]*∗*	14.2 (T2)

*∗* means statistically significant; † see Table 1; 1: acupuncture began 24 hours after surgery; 2: excluding Miles surgery; 3: incidence of medium to severe (grades II-III) abdominal distension.

CI: confidence interval; MD: mean difference.

## Supplementary Materials

Supplementary Materials Supp. 1: Search strategy used to identify studies of acupuncture and related therapies for CRC: PubMed. Figure S1: Funnel plot of acupuncture therapy versus postoperative care for time to first bowel sounds. Figure S2: Funnel plot of acupuncture therapy versus postoperative care for time to first flatus. Figure S3: Funnel plot of acupuncture therapy versus postoperative care for time to first defecation. Table S1: Risk of bias judgements for included studies. Table S2: Post hoc analyses of potential dose-response parameters for POI outcomes.
