# Supplementary material for: Acupuncture and Related Therapies for Treatment of Postoperative Ileus in Colorectal Cancer: A Systematic Review and Meta-Analysis of Randomized Controlled Trials
Source: Evid Based Complement Alternat Med. 2018 Jul 29;2018:3178472. doi: 10.1155/2018/3178472 (PMC6087601; doi:10.1155/2018/3178472)
Supplement: Supplementary Materials — Supp. 1: Search strategy used to identify studies of acupuncture and related therapies for CRC: PubMed. Figure S1: Funnel plot of acupuncture therapy versus postoperative care for time to first bowel sounds. Figure S2: Funnel plot of acupuncture therapy versus postoperative care for time to first flatus. Figure S3: Funnel plot of acupuncture therapy versus postoperative care for time to first defecation. Table S1: Risk of bias judgements for included studies. Table S2: Post hoc analyses of potential dose-response parameters for POI outcomes. [file 3178472.f1.pdf]

Acupuncture and related therapies for treatment of postoperative ileus in colorectal cancer: a systematic review and meta-analysis of randomized controlled trials

Yihong Liu[1], Brian H May[2], Anthony Lin Zhang[2], Xinfeng Guo[1], Chuanjian Lu[1] Charlie Changli Xue[1,2*], Haibo Zhang[1*]

[1]Guangdong Provincial Academy of Chinese Medical Sciences, Guangdong Provincial Hospital of Chinese Medicine, and The Second Clinical College, Guangzhou University of Chinese Medicine, Guangzhou, China
[2]China-Australia International Research Centre for Chinese Medicine, RMIT University, Bundoora, VIC 3083, Australia

Supplementary Materials

Supp 1 Search strategy used to identify studies of acupuncture and related therapies for CRC: PubMed

Terms used for Pub Med search (text word)
Group 1: Disease

colorectal cancer OR colorectal neoplasm OR colorectal carcinoma OR colonic cancer OR colon cancer OR colonic neoplasm OR colonic carcinoma OR rectal cancer OR rectal neoplasm OR rectal carcinoma OR large intestine cancer OR large intestine neoplasm OR large intestine carcinoma OR large intestinal cancer OR large intestinal neoplasm OR large intestinal carcinoma OR sigmoid cancer OR sigmoid neoplasm OR sigmoid carcinoma OR anal cancer OR anal neoplasm OR anal carcinoma OR CRC

Group 2: Intervention

acupuncture OR meridian OR electroacupuncture OR moxibustion OR auriculotherapy OR plum blossom OR acupressure OR ear-acupuncture OR ear-acupressure OR moxa OR laser acupuncture OR seven star needle OR acupoint OR electro-acupuncture OR electro acupuncture OR TENS OR transcutaneous nerve stimulation OR transcutaneous electric nerve stimulation OR transcutaneous electrical nerve stimulation OR electro-stimulation OR electro stimulation OR pharmacopuncture OR point injection OR catgut embedding OR dry-needling OR trigger point

Group 3: Study type

randomized controlled trial OR controlled clinical trial OR random OR randomized OR randomised OR randomly OR trial OR groups OR placebo OR sham OR systematic OR review OR meta-analysis OR metaanalysis

Combine

#Group 1

AND

#Group 2

AND

#Group 3

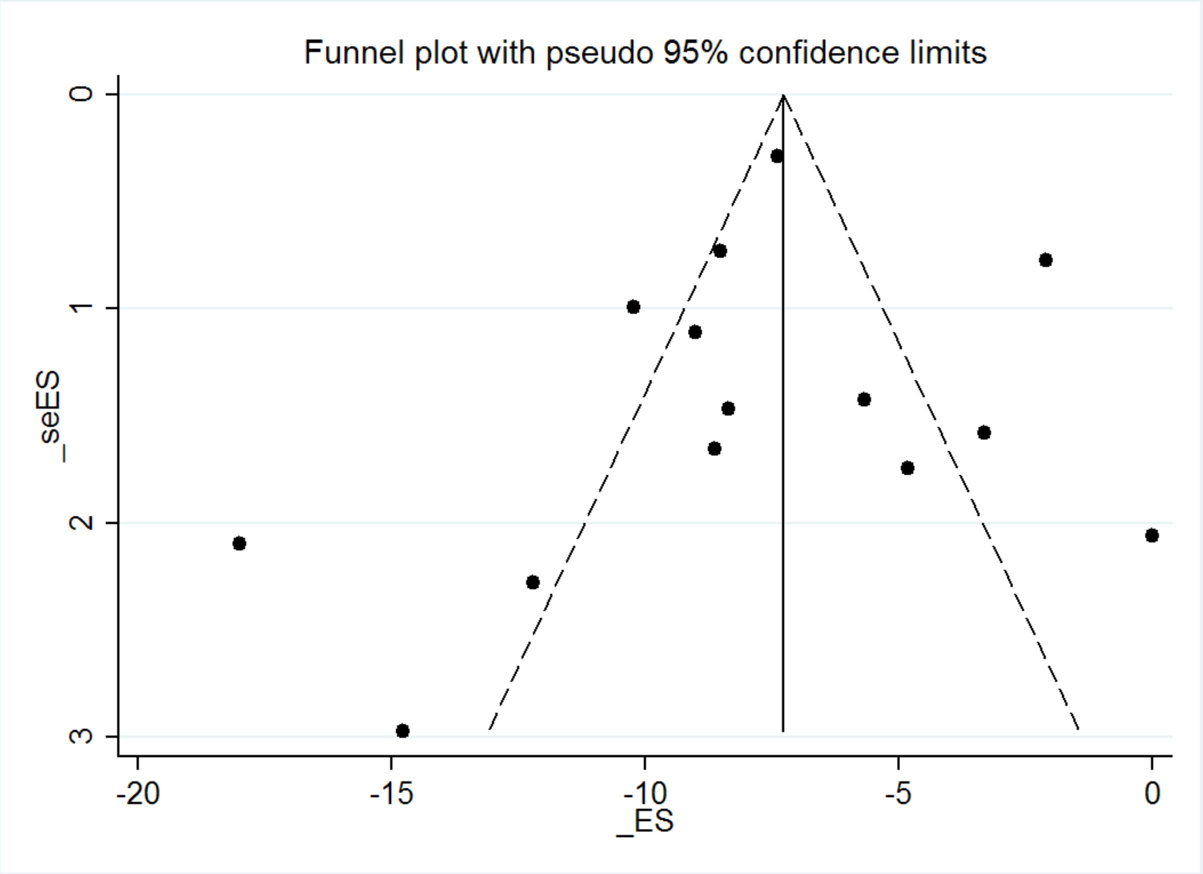

Figure S1 Funnel plot of acupuncture therapy versus post-operative care for time to first bowel sounds

Egger's test

Coef.= -0. 4969441, Std. Err.= 1.318204, t = -0.38, P>|t|= 0.713, 95% CI [-3.369063, 2.375175]

Not significant

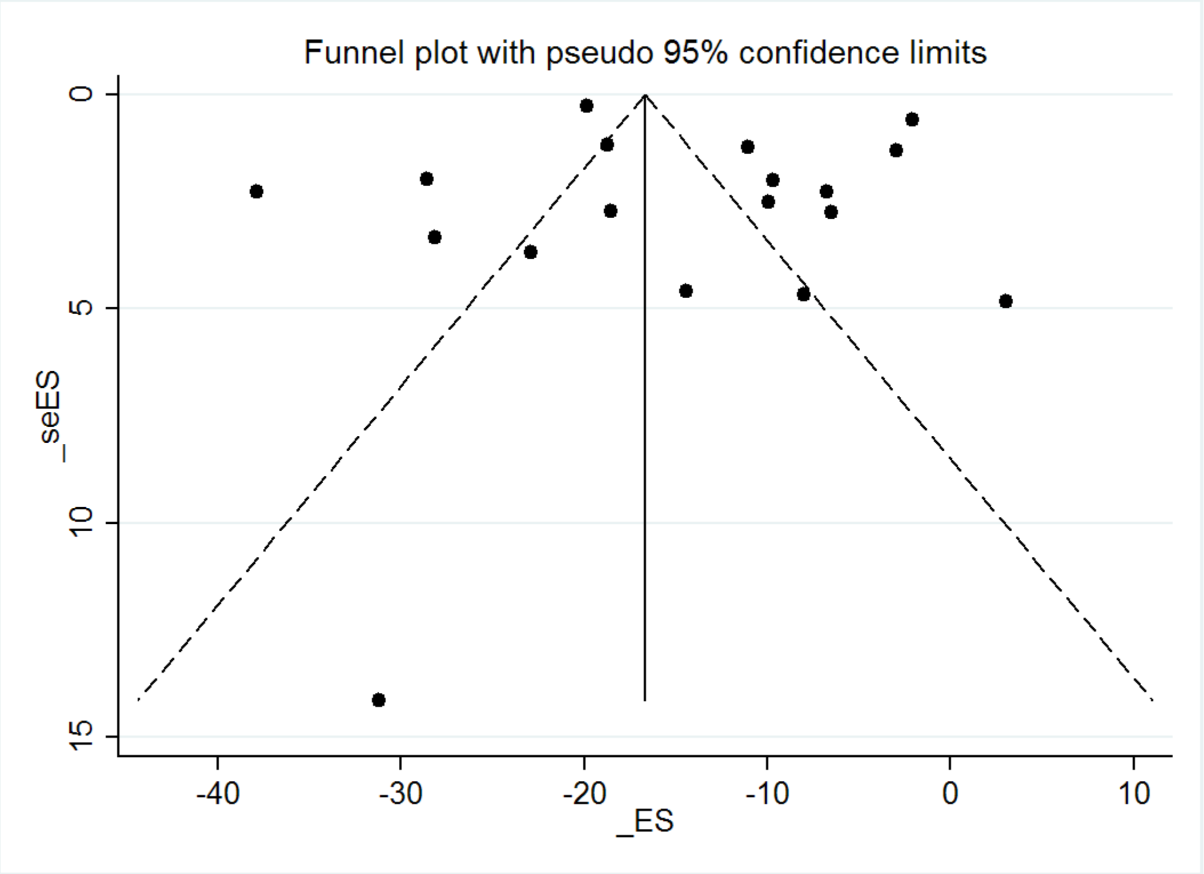

Figure S2 Funnel plot of acupuncture therapy versus post-operative care for time to first flatus

Egger's test

Coef.= 2.136238, Std. Err.= 2.43342, t = 0.88, P>|t|= 0.393, 95% CI [-3.022382, 7.294857]

Not significant

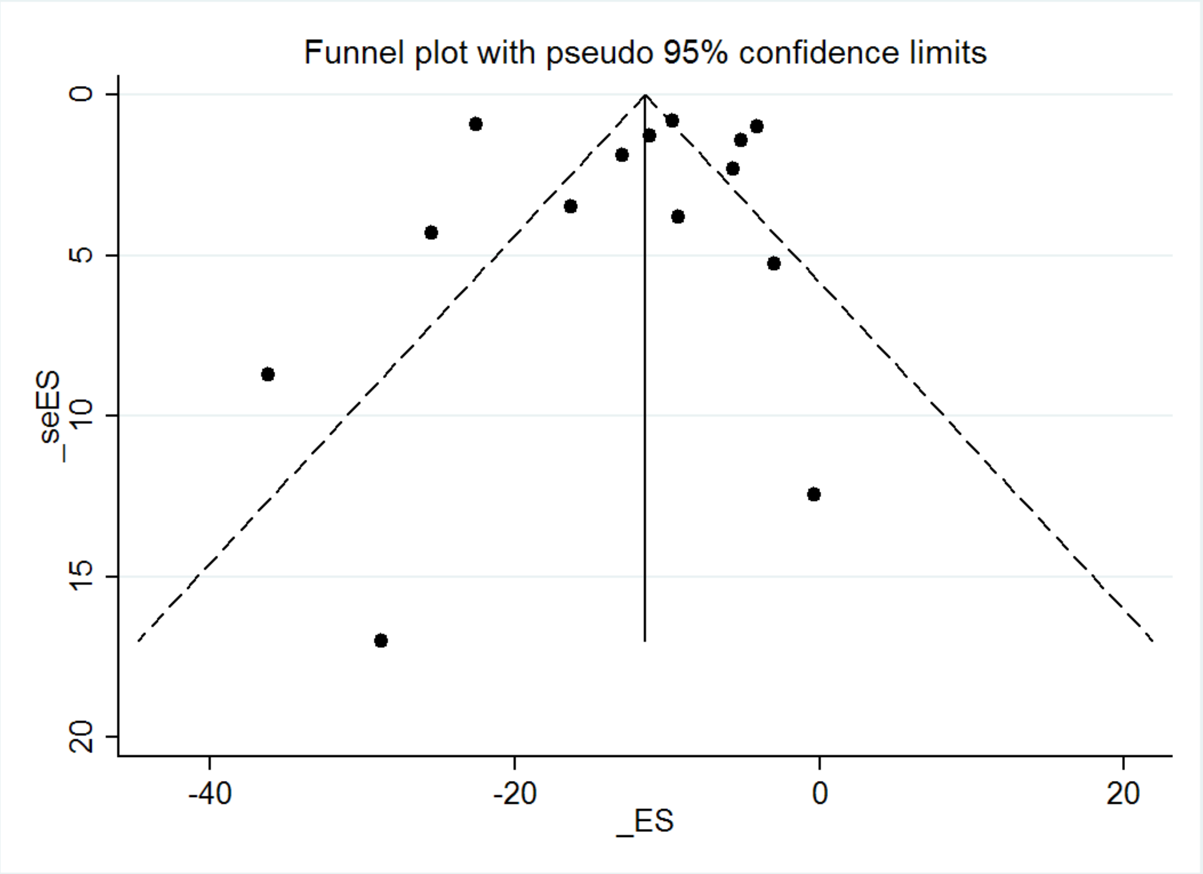

Figure S3 Funnel plot of acupuncture therapy versus post-operative care for time to first defecation

Egger's test

Coef.= -0. 3021557, Std. Err.= 2.030256, t = 0.15, P>|t|= 0.884, 95% CI [-4.725703, 4.121391]

Not significant

Table S1 Risk of bias judgements for included studies

Study ID	Author, year	SG	AC	BPt	BPn	BOA	IOD*	SOR
1	Chao HL *et al.*, 2013	U	U	U	H	L	L	U
2	Deng G *et al.*, 2013	L	L	L	H	L	L	L
3	Hong X 2017	H	U	H	H	H	L	U
4	Li JM *et al.*, 2016	U	U	H	H	H	L	U
5	Lu JY & Jin HM 2011	H	U	H	H	H	L	U
6	Meng ZQ *et al.*, 2010	L	U	H	H	H	L	U
7	Ng SSM *et al.*, 2013	L	L	L	H	L	L	L
8	Niu CF *et al.*, 2008	U	U	H	H	H	L	U
9	Si JG & Ding RS 2015	H	U	H	H	H	L	U
10	Tan S & Zheng CM 2015	U	U	H	H	H	L	U
11	Tong WY *et al.*, 2014	U	U	H	H	H	L	U
12	Wang EM 2012	L	U	H	H	H	L	U
13	Wang HM 2011	L	U	H	H	H	L	U
14	Xiao C 2014	L	U	H	H	H	L	U
15	Xiao L *et al.*, 2016	L	L	H	H	L	L	U
16	Yan YB 2011	U	U	H	H	H	L	U
17	Yang JJ *et al.*, 2011	L	U	H	H	H	L	U
18	Yang JF *et al.*, 2016	L	U	H	H	H	L	U
19	Zhang SY & Du YQ 2011	L	U	H	H	H	L	U
20	Zhang XY & Lu JB 2017	L	U	H	H	H	L	U
21	Zhang ZD *et al.*, 2014	L	L	L	H	L	L	U
22	Zhang XY *et al.*, 2016	L	U	H	H	H	L	U
	Totals	13 L, 3 H	4 L, 0 H	4 L, 17 H	0 L, 22 H	5 L, 17 H	22 L, 0 H	2 L, 0 H

Risk of Bias Categories: SG: Sequence Generation, AC: Allocation Concealment, BPt: Blinding of Participants, BPn: Blinding of Personnel, BOA: Blinding of Outcome Assessment, IOD: Incomplete Outcome Data, SOR: Selective Outcome Reporting.

Risk of Bias Judgements: L: low risk, U: Unclear risk or no information specified, H: High risk

Notes:
1. The difference in dropout rate between treatment and control groups did not exceed 20% in any study.
2. Only No 2 (Deng G *et al.*, 2013) and 7 (Ng SSM *et al.*, 2013) had an available protocol

Table S2 Post-hoc analyses of potential dose-response parameters for POI outcomes

Outcome	Subgroup (No. participants)	Acupuncture therapy	Effect Size MD [95% CI] I^2	Included Studies
Time to first bowel sounds (hours)	4-6 points (230)	All acup., acupressure, moxa on trad. points[1]	-7.00 [-10.45, -3.56]* 80.5%	14, 17, 20
	8-12 points (224)	All acup., acupressure, moxa on trad. points[1]	-9.31 [-15.08, -3.54]* 93%	9, 11, 13, 19
	Deqi (524)	All acupuncture therapies	-7.60 [-9.17, -6.04]* 65.0%	5, 10, 12, 14, 17, 20, 22
Time to first flatus (hours)	4-6 points (262)	All acup., acupressure, moxa on trad. points[1]	-11.44 [-18.35, -4.53]* 93%	8, 14, 17, 20
	8-12 points (469)	All acup., acupressure, moxa on trad. points[1]	-18.94 [-29.07, -8.82]* 93.6%	6, 7, 9, 11, 13, 15, 19
	Deqi (694)	All acupuncture therapies	-10.84 [-14.88, -6.79]* 96.8%	5, 7, 10, 12, 14, 15, 17, 20, 22
Time to first defecation (hours)	4-6 points (230)	All acup., acupressure, moxa on trad. points[1]	-10.91 [-15.78, -6.03]* 96.7%	14, 17, 20
	8-12 points (430)	All acup., acupressure, moxa on trad. points[1]	-17.34 [-25.78, -8.90]* 77.1%	6, 7, 11, 13, 15, 19
	Deqi (540)	All acupuncture therapies	-9.32 [-12.11, -6.53]* 72.2%	7, 12, 14, 15, 17, 20, 22

* Statistically significant; 1. excluding studies that used ear points.

Abbreviations: CI, confidence Interval; MD, mean difference; moxa, moxibustion; trad., traditional.
